# Reading More than Histones: The Prevalence of Nucleic Acid Binding among Reader Domains

**DOI:** 10.3390/molecules23102614

**Published:** 2018-10-12

**Authors:** Tyler M. Weaver, Emma A. Morrison, Catherine A. Musselman

**Affiliations:** Department of Biochemistry, Carver College of Medicine, University of Iowa, Iowa City, IA 52242, USA; tyler-weaver@uiowa.edu (T.M.W.); emma-morrison@uiowa.edu (E.A.M.)

**Keywords:** chromatin, histone, DNA, RNA, bromodomain, chromodomain, Tudor, PWWP, SANT, PHD finger

## Abstract

The eukaryotic genome is packaged into the cell nucleus in the form of chromatin, a complex of genomic DNA and histone proteins. Chromatin structure regulation is critical for all DNA templated processes and involves, among many things, extensive post-translational modification of the histone proteins. These modifications can be “read out” by histone binding subdomains known as histone reader domains. A large number of reader domains have been identified and found to selectively recognize an array of histone post-translational modifications in order to target, retain, or regulate chromatin-modifying and remodeling complexes at their substrates. Interestingly, an increasing number of these histone reader domains are being identified as also harboring nucleic acid binding activity. In this review, we present a summary of the histone reader domains currently known to bind nucleic acids, with a focus on the molecular mechanisms of binding and the interplay between DNA and histone recognition. Additionally, we highlight the functional implications of nucleic acid binding in chromatin association and regulation. We propose that nucleic acid binding is as functionally important as histone binding, and that a significant portion of the as yet untested reader domains will emerge to have nucleic acid binding capabilities.

## 1. Introduction

### 1.1. Chromatin and Reader Domains

The eukaryotic genome exists in the cell nucleus in the form of chromatin. The basic subunit of chromatin is the nucleosome, a complex of ~147 base pairs (bp) of DNA wrapped around an octamer of histones H2A, H2B, H3, and H4. Chromatin structure is extensively remodeled throughout the life-cycle of a cell. Such remodeling is important for all DNA-templated processes, and is critical in development and in response to external signals. An important facet of chromatin structure regulation is the post-translational modification (PTM) of the histone proteins. Histone PTMs, specifically acetylation and methylation of lysine, were originally identified in 1964 [1]. However, it was the discovery of the corresponding signaling machinery ~35 years later that propelled the field of epigenetics. Namely, the discovery of an acetyltransferase that catalyzes the placement of an acetyl group on lysine, a deacetylase that catalyzes removal of the acetyl group, and a bromodomain, which can specifically recognize acetylated lysine, revealed the signaling potential of histone PTMs [2,3]. 

Since then, a large number of histone PTMs have been discovered along with a plethora of protein domains that can specifically recognize them [4,5,6,7,8]. The latter are now collectively referred to as histone reader domains. There are several families of reader domains, which are classified based on structural fold, with each family recognizing a distinct PTM or small subset of PTMs. For example, bromodomains recognize a number of acetylated lysines [9], whereas chromodomains recognize only histone H3 di- or tri-methylated at lysine 9 (H3K9me2/3) or lysine 27 (H3K27me2/3) [10]. A large number of structural studies have revealed the mechanisms of histone recognition and specificity. However, it has proven extremely difficult to investigate the interaction of reader domains with the full nucleosome. Thus, the standard approach has been to investigate these interactions in the context of peptide fragments of the histones. Though these studies have revealed a great deal about PTM specificity, they preclude the ability to identify contacts outside the histone tails that might contribute to chromatin association. In recent years, however, a growing subset of known histone reader domains have been shown to also interact with DNA and/or RNA. This function has been known for a handful of chromodomains and PWWP domains for some time [10,11], but is now being discovered in an ever-growing number of histone readers. In addition, several studies have now confirmed the functional importance of this histone-independent activity in chromatin regulation. Here we review what is known about the mechanism and function of reader domain nucleic acid binding.

### 1.2. General Mechanisms of Nucleic Acid Binding

Protein-DNA binding has been studied for decades, and a wealth of information is available about the mechanisms of recognition by many DNA binding proteins. There are a number of protein folds known to bind double-stranded (ds) DNA [12]. The association of these domains with DNA can be mediated through interaction with the major and minor grooves, as well as the phosphate backbone. Specificity is generally classified as arising from two mechanisms: sequence specific readout consisting of direct or water-mediated contacts between amino acid side-chains and the bases, and shape readout, in which the contacts are mediated by a specific DNA architecture [12]. The latter includes DNA bending, kinking, and narrowing or widening of the grooves that are dependent on sequence. Notably, both mechanisms can be utilized by a single domain, and the two are often dependent on one another. There are also well characterized single-stranded (ss) DNA-binding domains [13]. Far fewer folds have been identified to bind to ssDNA as compared to dsDNA. Similar to dsDNA binding, the binding of ssDNA can be either sequence specific or non-specific. However, distinguishing these mechanisms can be difficult from structure alone, as both can involve direct readout of the bases in addition to phosphate backbone contacts. Thus, a thermodynamic assessment of selectivity is critical. Mechanisms of RNA binding and RNA binding domains are far more diverse, which mirrors the greater diversity in RNA structure and conformational flexibility [14]. RNA binding can be either sequence specific or non-specific. Due to the structural heterogeneity of RNA, aspects of all of the abovementioned DNA binding mechanisms are observed in RNA binding. 

Strength of binding is assessed through measurement of the in vitro dissociation constant (K_d_). These can be very roughly categorized as strong (picomolar-nanomolar range), moderate (micromolar range), or weak (millimolar range). dsDNA-binding domains are often seen to associate in the picomolar (pM) to low micromolar (μM) range [15,16]. Although dsDNA-binding domains that demonstrate sequence-specific binding can also associate non-specifically with weaker affinity. In general, in vitro RNA-binding affinities and ssDNA-binding affinities are weaker than dsDNA-binding affinities, often in the micromolar range, though there are exceptions that bind much tighter.

## 2. Overview and Importance in Function

### 2.1. Prevalence of Nucleic Acid Binding among Histone Readers and Interplay with Histone Binding

Currently, members of the bromodomain (BD), PHD finger, PWWP, chromodomain (CD), Tudor, and SANT/Myb families of reader domains have been identified as harboring the ability to bind ssDNA, dsDNA, ssRNA, or dsRNA, as seen in Table 1. Thus, 6 of the 23 families of known reader domains have members that have been identified as harboring nucleic acid-binding activity. Depending on the specific domain, the nucleic acid-binding activity may be independent from the histone-binding activity, and in some cases it is found to actually enhance histone binding. Conversely, in a few cases histone binding enhances association with nucleic acid. Currently there are no cases where binding to histone or DNA precludes association with the other substrate. While binding to histone peptides ranges from low micromolar to millimolar affinity, binding to nucleic acid is generally higher affinity, ranging from nanomolar to high micromolar. However, there are exceptions to this, such as the PHF1 Tudor, 53BP1 TTD, mCBX7 CD, and BRDT1 BD1, in which histone binding actually dominates between the two activities [17,18,19,20,21,22]. 

### 2.2. Comparison to Previously Characterized Nucleic Acid-Binding Domains

Comparison of binding affinities between nucleic acid binding domains is notoriously difficult as differences in experimental conditions and sequence length can substantially alter measured binding constants. However, as a rough comparison, most of the reader domains currently characterized associate with moderate affinity to nucleic acids. They generally bind dsDNA weaker than previously characterized DNA-binding domains, while the RNA binding is comparable to known RNA-binding domains. Weaker affinity to dsDNA could be because the nucleic acid-binding is only one contact in a multivalent mechanism of chromatin association. Indeed, for several domains, it is the combination of histone and DNA binding that leads to high affinity interaction with nucleosomes. It could also be because the highest affinity substrates have not yet been identified. Currently, only a very limited number of substrates has been tested. For many domains, even a comparison between RNA and DNA binding has not yet been carried out. 

To date, little sequence specificity has been observed, either via direct sequence readout or shape readout. However, it should be noted that only very limited sequence space has been explored. At best, only AT-rich versus GC-rich sequences have been compared for most domains. The two exceptions to this are the HDGF PWWP domain and the cMyb SANT/Myb repeats, both of which have been found to have sequence specificity [31,63,64,65,66,67]. Finally, though there is substantial information on the nucleic acid-binding interfaces provided by nuclear magnetic resonance (NMR) spectroscopy studies or mutagenesis, there is currently a dearth of atomic-level structures of these complexes. Thus, the mechanism by which most of these domains associate with nucleic acids is not fully clear. 

### 2.3. Functional Implications

The interactions between reader domains and nucleic acids, which are discussed in detail in the next section, have a variety of mechanistic and functional implications for the parent chromatin regulatory protein or complex. While the nucleic acid binding function of many of these domains remains untested or unclear, a few have been investigated. Based on this limited data some mechanisms of function are emerging. In addition to these, there are a number of other potential, non-mutually-exclusive mechanisms of function for the nucleic acid-binding properties of these domains that are discussed below. 

**Figure 1:**
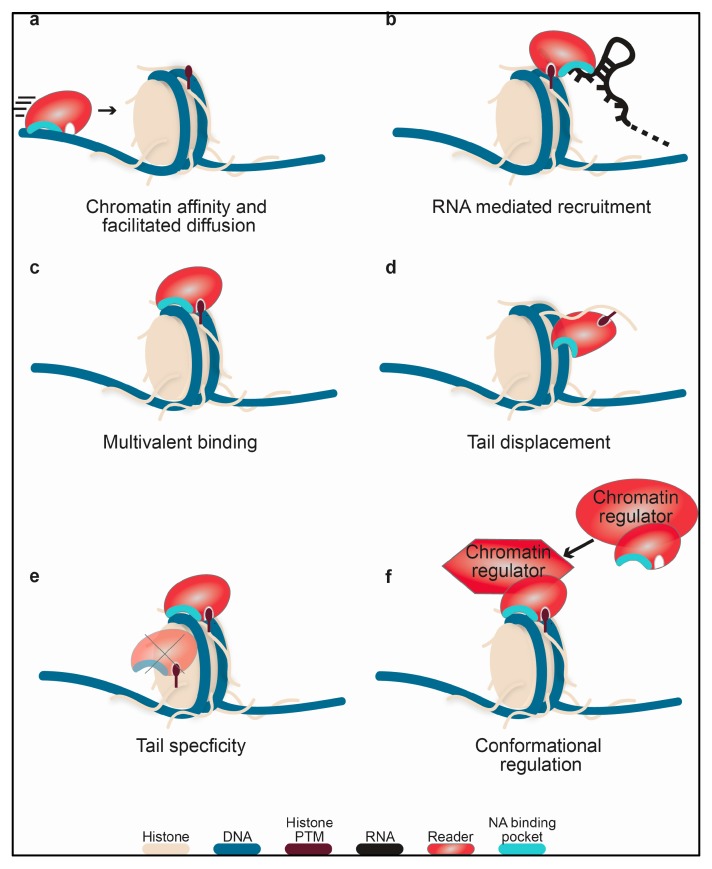
Models of the functional consequence of nucleic acid binding. Shown for each is a cartoon representation of a mono-nucleosome (DNA dark blue, histones wheat), containing a histone post-translational modification (PTM) (crimson oval), and a reader domain (red) containing a PTM binding pocket and a DNA binding surface (cyan). Representations of nucleic acid binding contributing to (**a**) chromatin affinity and/or facilitated diffusion, (**b**) RNA mediated recruitment, (**c**) multivalent nucleosome binding, (**d**) histone tail displacement from DNA, (**e**) histone tail specificity through positioning, or (**f**) modulation of regulator activity through conformational change.

Currently, the best characterized function of reader domain nucleic-acid binding is a substantial contribution to the affinity of the host protein/complex for chromatin and/or retention of it at target sites as seen in Figure 1a,b. This includes direct association with chromatin DNA, as well as indirect association via interaction with RNA. Though not currently tested, this could include a role in facilitated diffusion along DNA [68]. This would reduce the search space dimensionality and allow the protein/complex to more efficiently sample the chromatin landscape, as proposed for BRDT BD1 [17]. Notably, the effective contribution of the reader domain to the chromatin affinity will depend largely on the presence of other DNA-binding domains in the complex. For instance, the BRG1/BRM BDs are not seen to contribute substantially to BAF affinity for bulk chromatin [23], perhaps not surprising as this megadalton complex contains several other DNA-binding domains. 

For several reader domains, the nucleic acid-binding activity, coupled with the histone-binding activity, has been shown to lead to increased affinity for nucleosomes through multivalent contacts, as seen in Figure 1c. This was observed for BRDT BD1 [17], the Pdp1 and PSIP1 PWWP domains [34], the PHF1 Tudor domain [20], and the ZMYND11 integrated domain [69]. Notably, carrying a DNA- and histone-binding interface on a single domain is thermodynamically advantageous, as this minimizes the loss in conformational entropy that occurs upon binding when these binding pockets occur on two flexibly linked domains. 

Association of a reader domain with DNA could potentially act to displace histone tails from linker or nucleosomal DNA as seen in Figure 1d. The N-terminal tails of histones have been known to interact with DNA for decades [70]. Recently, studies have shown that this interaction can inhibit reader and writer activity towards nucleosomes [71,72,73]. In particular, these studies reveal that accessibility of the histone tails to readers and writers was occluded within the nucleosome. Reader domain binding to DNA could therefore increase the accessibility of histone tails and enhance downstream activity. This type of tail-displacement mechanism has been proposed for the LSD1-CoREST heterodimeric enzyme complex, wherein the SANT2 domain of the CoREST monomer interacts with nucleosomal DNA, displacing the H3 tails from the nucleosome core and making them available for interaction with the LSD1 catalytic core [60]. In this example, the DNA- and histone-binding arise from separate protein subunits, but a similar mechanism could be envisioned for DNA- and histone-binding interfaces on two domains within the same protein or two binding interfaces on the same domain. 

Nucleic acid-binding ability may also provide specificity for a given histone residue as shown in see Figure 1e. For BRDT BD1, it was proposed that DNA binding contributes to acetyl-lysine specificity within the context of the nucleosome by properly positioning the domain with respect to the histone PTM [17]. Similarly, adjacent DNA- and histone-binding pockets could enhance specificity for a histone modification positioned close to the nucleosome core, such as H3K36, which is positioned proximal to where the H3 tail protrudes from the nucleosomal DNA. Nucleic acid binding may also be necessary in order for a reader domain to associate with histone tails, or vice-versa. For instance, the hMSL-3 Chromobarrel was shown to robustly associate with H4K20me1 peptide only in the presence DNA [37]. Conversely, the scClr4 CD can only interact with ssRNA, ssDNA and dsDNA in the presence of H3K9me2 [46]. 

Finally, association of a reader domain with nucleic acid could be involved in regulating the activity of the parent protein or complex. Potential mechanisms for this include proper positioning of the active site at the nucleosomal substrate or an induced conformational change in the complex itself such as ordering of an enzymatic pocket or relief of auto-inhibition, as seen in Figure 1f. Alternatively, or additionally, binding could induce a conformational change in the nucleosome. Recently, single molecule studies have demonstrated that histone tail deletion alters the energy landscape of nucleosome remodeling [74], and thus histone tail displacement could positively impact chromatin remodeling. In addition, binding of the PHF1 Tudor domain to the nucleosome was shown to alter the breathing dynamics of the DNA [20]. Though the exact functional effect of this is still not known, it is easy to envision how such a perturbation could alter regulator function. 

## 3. Nucleic Acid-Binding Reader Domains

Below, we review the specific reader domains for which nucleic acid binding has been identified. As several previous reviews have discussed the mechanisms of histone binding of these domains, we only briefly mention their cognate targets. When possible, we compare DNA and histone binding, though we note that such comparisons can be challenging due to differences in experimental technique and conditions. We describe in detail the structural mechanisms of DNA binding, and the functional importance of these interactions, for those that are known. Information is organized by reader domain family. 

### 3.1. Bromodomains

**Figure 2:**
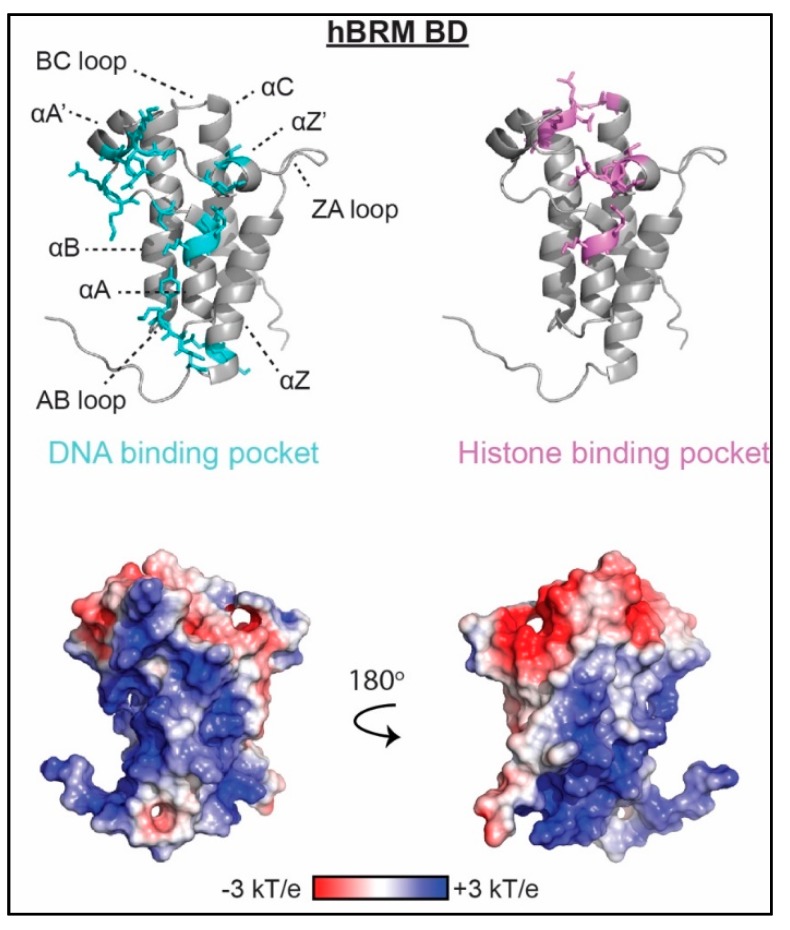
The molecular mechanism of bromodomain (BD) association with nucleic acid. Residues determined to be important for binding nucleic acid as mapped by nuclear magnetic resonance (NMR) spectroscopy and/or mutagenesis are shown as cyan sticks on ribbon representations of the BRM BD structure (PDBID 2DAT). Residues important for histone binding are shown as violet sticks. Corresponding representation of the electrostatic surface (determined using the APBS plug-in in pymol) is shown in two orientations.

Bromodomains are well-characterized acetyl-lysine binding domains that can associate with histone and non-histone proteins. Specificity for a particular acetylated lysine is protein dependent, with some BDs displaying a high degree of specificity and others demonstrating promiscuous binding [9]. The BD structure is broadly conserved and consists of ~120 residues forming a left-handed bundle of four α-helices connected by several variable-length loops that form the acetyl-lysine binding pocket [9] as seen in Figure 2. The potential for interactions between a BD and DNA was first proposed by the Tijan laboratory in 2000 [75], however it was over 15 years later before an interaction was experimentally observed. Recently, several BDs from the human BET family (BRDT BD1, BRD2 BD1 and BD2, BRD3 BD2 and BRD4 BD2) as well as the human BRM and homologous BRG1 BDs have been found to bind DNA [17,23]. In addition, the BRD2, BRD3, BRD4, and BRD7 tandem BDs were recently shown to bind RNA [76]. There is currently no apparent sequence specificity, though only a small subset of sequences has been tested for each BD. The molecular mechanism of binding has only been described for BRDT BD1 and BRM/BRG1 BD association with DNA. 

Binding pockets for DNA or nucleosomes were mapped using NMR spectroscopy and reveal interaction surfaces that are rich in arginine and lysine residues. For both BDs, the DNA-binding pockets are seen to be largely non-overlapping with the histone-binding pockets. However, unlike the acetyl-lysine binding pocket, which is highly conserved, the composition and location of the DNA binding surface is not shared between the BDs. BRDT BD1 associates with DNA via a basic patch centered on the αZ helix whereas the surface basic patch of the BRM/BRG1 BD is comprised of residues in the αA helix, the ZA and AB loops, and the very N-terminal end of the αZ helix, as seen in Figure 2. The BD1 of BRDT, which associates with H4 acetylated at lysine 5 and lysine 8 (H4K5acK8ac), binds a 25 bp dsDNA with an apparent dissociation constant (K_d_) of 52 μM at 100 mM NaCl. This is weaker than the histone binding at K_d_ = 13 μM in 150 mM NaCl. In contrast, the BRM BD binds more weakly with K_d_ = 600 μM for a 10 bp DNA and K_d_ = 900 μM for an H3K14ac peptide, both at 50 mM KPi and 50 mM KCl. 

Both BDs can bind histones and DNA contemporaneously. The binding events are not allosterically linked, but provide the potential to multivalently associate with nucleosomes. This has been demonstrated for BRDT BD1, where association with the nucleosome leads to an affinity of K_d_ = 2 μM at 150 mM NaCl, substantially stronger than either histone tail or DNA alone. Interestingly, the position of the histone tail within the context of the nucleosome was important for BRDT BD1 specificity, as a chimera with the acetylated H4 tail attached to the H3 core rather than the H4 core led to decreased affinity for the BD. It was proposed that DNA binding might play a role in positioning the domain with respect to the histone tail, thus contributing to the observed specificity. However, conformational differences in the tail itself could also be playing a role. Mutating DNA-binding residues in BRDT BD1 leads to decreased affinity for bulk chromatin and defective chromatin compaction in response to hyperacetylation, indicating that association with DNA is important for chromatin association and BRDT function. In contrast, disrupting the DNA binding ability of the BRG1 BD in mouse embryonic stem cells does not affect the global chromatin affinity of BRG1, suggesting a more nuanced regulatory role in BAF function [23].

Most recently, the tandem BDs of BRD4 (BD1/2) were found to associate with enhancer RNA (eRNA). The molecular mechanism of binding and affinity for RNA have not yet been determined. However, the linked BDs were found to be necessary for association of BRD4 with eRNA in vitro. Furthermore, in vivo, BRD4 was found to be important for eRNA synthesis and its subsequent association was found to be critical for transcription of the associated mRNA, in a manner dependent on the BDs [76]. Notably, in vitro association of BRD2, BRD3, and BRD7 BD1/2 with eRNA were also observed, as well as weaker binding of BRDT BD1/2 and BRG1 BD. 

### 3.2. PHD Fingers

**Figure 3:**
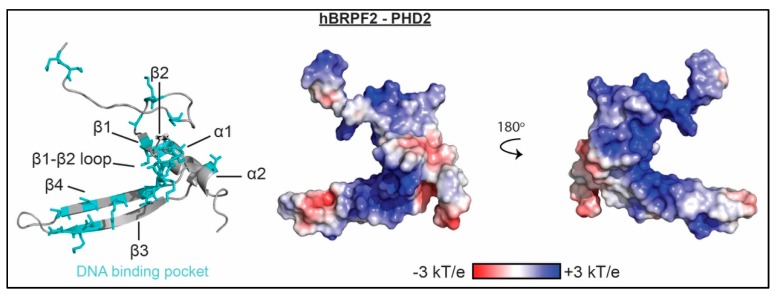
The molecular mechanism of PHD association with nucleic acid. Residues determined to be important for binding nucleic acid as mapped by NMR spectroscopy and/or mutagenesis are shown as cyan sticks on a ribbon representation of the BRPF2 PHD2 structure (PDBID 2LQ6). Corresponding representation of the electrostatic surface (determined using the APBS plug-in in pymol) is shown in two orientations.

PHD fingers typically associate with the histone H3 tail, with most PHD fingers specific for either unmodified or trimethylated H3K4 (H3K4me0 or H3K4me3) [77]. PHD fingers are zinc-binding domains with a canonical C_4_HC_3_ motif that coordinates two Zn^2+^ ions on either side of a two-stranded anti-parallel β-sheet. This is often followed by a small C-terminal α-helix. Though no PHD fingers have been identified that associate with both histones and nucleic acid, two PHD fingers have been identified that lack histone binding activity but bind to DNA. These two PHD fingers are both structurally atypical, coordinating the two Zn^2+^ ions via a C_3_HC_2_H motif and harboring an additional two-stranded anti-parallel β-sheet (see Figure 3). However, the mode of binding DNA is not conserved between them.

BRPF2 contains an N-terminal PHD finger-Zn knuckle-PHD finger (PZP) module. While the first PHD finger of human BRPF2 associates with H3K4me0, the second PHD finger in this module does not associate with histones and instead has been found to bind DNA. PHD2 has an additional, extended two-stranded anti-parallel β-sheet (β3 and β4), and lacks the conserved histone binding residues as compared to canonical PHD fingers. NMR and mutagenesis studies revealed that histidine, lysine, and arginine residues in the β1-β2 loop, and β3 and β4 strands are involved in interactions with DNA as seen in Figure 3. 

PHF6 also contains an atypical PHD finger. The second extended PHD finger (ePHD2) of human PHF6 consists of an N-terminal zinc finger that connects to an atypical PHD finger via a long linker. Together, the zinc finger, linker, and PHD finger form an integrated folded unit. Similar to BRPF2 PHD2, the PHD component of the PHF6 ePHD2 has an additional two-stranded β-sheet. The PHD finger lacks the conserved histone binding residues, and an α-helix within the linker region occludes what would be the normal histone binding surface. This module was shown not to bind histones as expected, but was instead found to bind DNA. NMR spectroscopy was used to map the binding interface. The zinc finger as well as residues in the linker α-helix and N-terminal region of the PHD finger were found to be involved in binding, and mutagenesis confirmed the importance of basic residues in the zinc finger and linker region in associating with DNA. 

Both associate with dsDNA in the low micromolar range, K_d_ = 4 μM for BRPF2 PHD2 and K_d_ ≈ 13 μM for PHF6 PHD2 (both at 100 mM NaCl) [26,27]. Among the sequences tested, no preference for AT- or GC-rich DNA was observed [26,27]. The function of the DNA-binding activity of these PHD fingers is currently unknown. 

### 3.3. PWWP Domains

**Figure 4:**
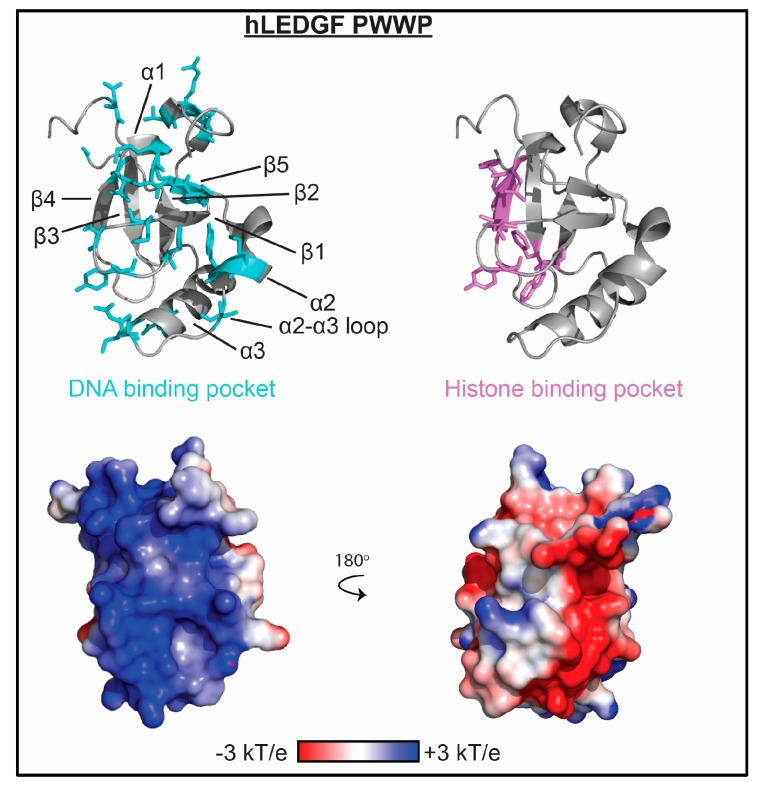
The molecular mechanism of PWWP association with nucleic acid. Residues determined to be important for binding nucleic acid as mapped by NMR spectroscopy and/or mutagenesis are shown as cyan sticks on ribbon representations of the LEDGF PWWP structure (PDBID 4FU6). Residues important for histone binding are shown as violet sticks. Corresponding representation of the electrostatic surface (determined using the APBS plug-in in pymol) is shown in two orientations.

The PWWP domain recognizes methylated-lysine, and to date all PWWP domains have been found to specify for either H3K36me3, H3K79me3, or H4K20me3. The PWWP domain structure consists of a five-stranded anti-parallel β-barrel followed by a variable helical bundle of between one and six α-helices as seen in Figure 4. PWWP domains contain a loosely conserved namesake proline-tryptophan-tryptophan-proline motif in the β2 strand, which packs against the helical bundle. Notably, the DNA-binding ability of PWWP domains was experimentally determined before the histone binding function was discovered [78,79,80,81], as has been previously been reviewed in [11]. There are now several PWWP domains known to bind to dsDNA. To date this includes the Dnmt3b, *S. pombe* Pdp1, hepatoma derived growth factor (HDGF), MSH6, and PSIP1 (also called LEDGF or p75) PWWP domains [29,30,31,33,34,35,78,82]. Binding affinities for DNA are strong to moderate with K_d_ values ranging from 0.13–150 μM and most associating with K_d_ < 10 μM. This contrasts with the generally weak association seen for methylated histone peptides, with most PWWPs displaying K_d_ values in the millimolar range [29,32,34,35]. As with most reader domains, sequence preference of the majority of the PWWP domains has not been thoroughly explored. However, an in vitro selected and amplified binding assay (SAAB) determined that DNA binding by the *Rattus norvegicus* HDGF PWWP is largely non-specific, with only a weak preference for CACC elements [30]. In a separate study, a ChIP-based assay found that human HDGF specifically targets a sequence within the promoter region of the *SMYD1* gene, containing a GACC element, and that the PWWP domain is necessary and sufficient for this binding [31]. 

Unlike PHD fingers and BDs, the mode of DNA binding appears to be largely conserved among PWWP domains for which structural data is available. Primarily NMR spectroscopy studies have demonstrated that the DNA binding pocket consists of a surface basic patch adjacent to the methyl-lysine binding pocket. In general, this includes the β1-β2 arch region (which includes the poorly conserved PWWP motif) and portions of the helical region immediately following the final β-strand as seen in see Figure 4. Depending on the PWWP domain, other regions within the β-strand and helical regions may be involved. Recently, a crystal structure of the *Rattus norvegicus* HDGF PWWP was solved in complex with 10bp of the *SMYD1* promoter DNA sequence. Within this complex, the PWWP crystallized as a domain-swapped dimer, where residues 19–22 from one monomer and 77–80 of the second monomer interact with DNA [83]. The binding interface predicted from NMR-based studies includes these two regions, but extends to cover a wider portion of the surface basic patch. 

Except for the HDGF PWWP, for which histone binding has not yet been investigated, these PWWP domains have all been shown to bind methylated histone tails [29,32,34,35,82]. As the DNA and histone binding pockets do not overlap extensively, it suggests that binding to both ligands is possible, and PWWP domains may associate with nucleosomes in a multivalent manner. Indeed, the Pdp1 PWWP domain was found to bind to both H4K20me3 and DNA simultaneously [29], and there was no change in affinity for DNA in the presence of H4K20me3, indicating independent binding events. Binding of the Pdp1 PWWP domain to both unmodified nucleosomes and nucleosomes containing a methyl-lysine analog at H4K20 (H4K_C_20me3) were dependent on the DNA-binding residues. Though DNA binding was proposed to be the major driver in nucleosome association, a modest increase in affinity was observed for H4K_C_20me3-nucleosomes, which was proposed to lend selectivity for H4K20me3 within chromatin. On the other hand, selectivity for methylated nucleosomes is much stronger for the PSIP1 PWWP domain. The PSIP1 PWWP domain binds DNA with significantly higher affinity than H3K36me3 peptides [34,35]; however, binding to the H3K_C_36me3-nucleosome is greater than an order of magnitude tighter than to an unmodified nucleosome [34,35]. Mutations to arginine and lysine residues on the DNA-binding surface reduced binding to nucleosomes, but maintained specificity for the H3K_C_36me3-nucleosome [34]. Together, this suggests that interactions with both H3K36me3 and DNA drive specific, high-affinity association with the nucleosome. 

The mechanism by which PWWP DNA binding contributes to the function for these proteins remains elusive. However, in the case of Pdp1 and PSIP1, it is clear that this interaction is functionally important. Pdp1 regulates Set9-mediated methylation of H4K20 and is implicated in the DNA double-strand break repair pathway [81]. *S. pombe* lacking Pdp1 were unable to maintain levels of H4K20me2/me3, which was rescued upon re-expression of wild type Pdp1 but not Pdp1 carrying mutations to the DNA-binding surface of the PWWP domain [29]. PSIP1 is a co-factor of lentiviral DNA integration that stimulates HIV-1 integration by interacting with the viral integrase enzyme via the C-terminal region and chromatin via the N-terminal region [84]. Mutating the DNA binding pocket of the PSIP1 PWWP domain leads to a loss of this stimulation [85], supporting the biological relevance of the DNA binding ability. 

### 3.4. Chromodomains

Chromodomains (CDs) and chromo barrel domains (Chromobarrels) are histone reader domains that interact with mono-, di-, and tri-methylated lysine residues on the histone H3 and H4 tails (extensively reviewed in Reference [10,86]). CDs are small ~50 amino acid domains that consists of a three-stranded antiparallel β-barrel followed by a C-terminal α-helix (αA-helix), with the Chromobarrel containing an additional β-strand (β_0_) before the CD fold. Several CDs and Chromobarrels (note these are often mistakenly referred to as CDs) have been identified to associate with DNA and/or RNA. This includes the CDs from CBX2/4/6/7/8, SUV39H1 and the fission yeast homologue Clr4, Chp1, and Mi-2 [19,39,40,43,44,46]. In addition, the Chromobarrel domains from MOF and MSL3 [37,87] can associate with nucleic acids. All of these domains have been shown to bind methylated histone peptides [18,19,37,38,42,45] except for the Mi-2 CDs and the MOF CD, which lack the critical aromatic cage residues needed for methylated histone binding. MOF has been experimentally confirmed not to associate with methylated histone tails [36]. The SUV39H1, Clr4, and CBX CDs, as well as the MOF Chromobarrel, demonstrate preference for RNA over DNA (though moderate in some cases). In general, nucleic acid binding affinities of these domains range between K_d_ = 0.3 μM and K_d_ = 51 μM, which is comparable to the observed range of histone binding affinities (0.1 μM < K_d_ < 55 μM). In contrast to other reader domains, where the histone and nucleic acid binding is seen to be largely independent, there are instances where binding to nucleic acid increases histone affinity or vice versa [37,46]. 

**Figure 5:**
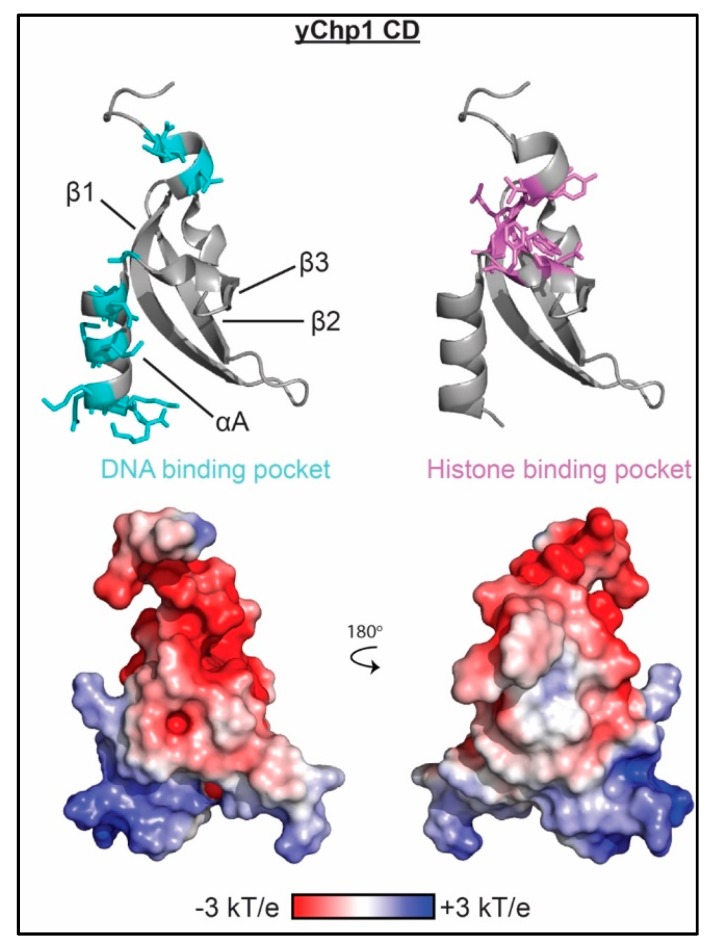
The molecular mechanism of CD association with nucleic acid. Residues determined to be important for binding nucleic acid as mapped by NMR spectroscopy and/or mutagenesis are shown as cyan sticks on ribbon representations of the Chp1 CD structure (PDBID 2RSN). Residues important for histone binding are shown as violet sticks. Corresponding representation of the electrostatic surface (determined using the APBS plug-in in pymol) is shown in two orientations.

The majority of the structural information on CD/Chromobarrel interaction with nucleic acids comes from NMR spectroscopy and mutagenesis studies. The SUV39H1, Clr4, and Chp1 CDs bind RNA through basic residues in the αA-helix, as seen in Figure 5 [43,44,46]. This interaction surface is distinct from the methyl-lysine binding pocket, but notably for Clr4 and Chp1, histone binding substantially increases nucleic acid affinity [46] by an unknown mechanism. The distinct binding pockets suggest the ability to bind both histones and nucleic acid. Recently, a cryo-EM structure of the Chp1 CD bound to H3K9me3-nucleocomes was solved to 7.3 Å resolution [88]. The resolution is not high enough to fully resolve the CD/histone interaction, however the histone binding pocket is oriented toward the H3K9me3 tail, and this orients the basic patch of the αA-helix away from the core of the nucleosome indicating that in the context of the nucleosome the CD could bind both H3K9me3 nucleosomes and RNA. 

Another mechanism of binding is observed for the CBX7 CD and MSL3 Chromobarrel [37,40]. These preferentially associate with RNA and dsDNA, respectively, through the β-barrel. CBX7 utilizes the β1, β2, and β3 strands and MSL3 utilizes the β3 and β4 strands and intervening loop. Here, the nucleic acid and histone binding pockets partially overlap, but a ternary complex has been shown to be formed by both. However, in the case of MSL3 there is evidence that DNA binding is in fact necessary for histone association. Whereas, one study found the dMSL-3 Chromobarrel binds all methylated states for H4K20 and H3K36, with preference for the mono- and di- over tri-methylated states [38], another study found that the dMSL-3 and hMSL-3 Chromobarrels do not interact with histone peptides alone, but rather require DNA binding to mediate this interaction, which specifically enables interaction with H4K20me1 [37]. This is thought to mediate specific interaction with the nucleosome, but this has not been tested. 

Some of these interactions appear to be important in RNA-mediated chromatin targeting. The drosophila MOF histone acetyltransferase is a part of the dosage compensation complex (DCC) that upregulates genes on the male X chromosome. Targeting of dMOF to the male X chromosome is mediated by interactions with the RNAs *roX1* and *roX2* [89]. Deletion of the Chromobarrel or mutation of conserved residues in the domain resulted in loss of RNA binding in vitro and loss of *rox2* RNA binding in vivo [87]. MSL3 is another member of the drosophila DCC, and its Chromobarrel was also shown to be important in DCC function. Specifically, deletion of the MSL3 Chromobarrel results in reduced affinity of dMSL-3 for RNA and DNA in vitro, mis-localization of the DCC in vivo, and significant up-regulation of dosage compensation genes on the male X chromosome [87,90,91,92]. The SUV39H1 histone methyltransferase catalyzes trimethylation of H3K9 and is important for establishing pericentric heterochromatin. Mutation of the hSUV39H1 CD RNA-binding pocket resulted in a significant decrease in SUV39H1 binding to α-satellite RNA and pericentric heterochromatin in human cell lines [44], as well as a decrease in the establishment and maintenance of pericentric heterochromatin in mouse embryonic fibroblasts [43]. Compromised centromeric silencing is also seen upon mutation of the RNA binding residues in the CD of the yeast homologue Clr4 [46]. Chp1 is yet another protein involved in the formation of centromeric heterochromatin in fission yeast, which acts through the RNA interference (RNAi) pathway. Mutation of the CD RNA-binding pocket resulted in decreased RNA binding compared to wildtype Chp1 as well as partially defective heterochromatin silencing [46]. 

For some of the other CDs, nucleic acid interaction is clearly critical in the function of the host protein/complex, though the exact mechanism remains elusive. The CBX proteins are members of the polycomb repressive complex 1 (PRC1) histone ubiquitin ligase complex, which is essential for developmental gene repression. RNA binding by the hCBX7 CD has been shown to be important for PRC1 complex repressive activity. Specifically, the INK4a/ARF locus, which is normally repressed by PRC1, becomes de-repressed when the RNA binding site is mutated [40]. Impaired function is also seen for the drosophila Mi-2 ATP-dependent chromatin remodeler. Specifically, deletion of the two CDs results in loss of linear and nucleosomal DNA binding by dMi-2, decreased ATPase activity, and impaired nucleosome sliding [47]. Notably, two endometrial cancer mutations (V558F and R572Q) in the human homologue CHD4 (which are conserved in dMi-2) have been identified in CD1 [93]. Both mutations result in decreased DNA binding and impaired ATPase/nucleosome remodeling by the dMi-2 protein.

### 3.5. Tudor Domains

**Figure 6:**
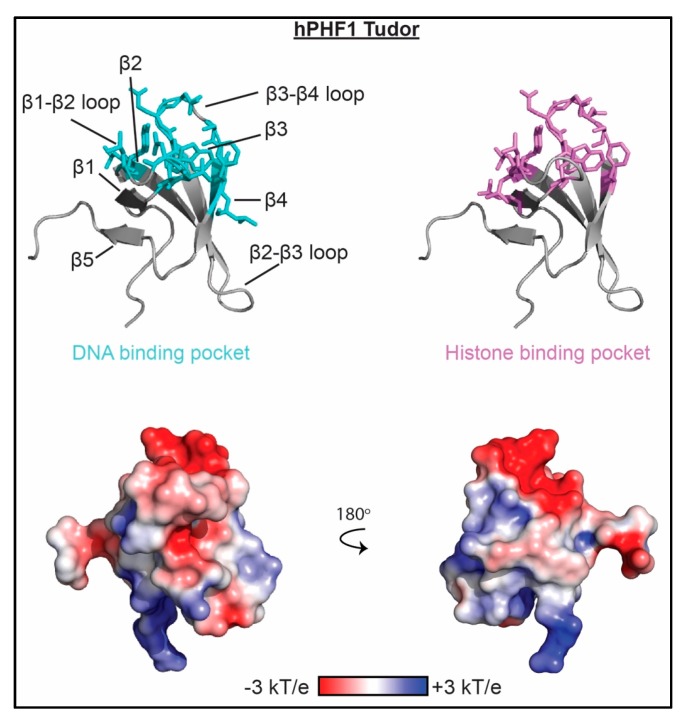
The molecular mechanism of Tudor association with nucleic acid. Residues determined to be important for binding nucleic acid as mapped by NMR spectroscopy and/or mutagenesis are shown as cyan sticks on ribbon representations of the PHF1 Tudor structure (PDBID 4HCZ). Residues important for histone binding are shown as violet sticks. Corresponding representation of the electrostatic surface (determined using the APBS plug-in in pymol) is shown in two orientations.

Tudor domains, Tandem-Tudor domains (TTD) and the knotted-Tudor are histone reader domains that specifically recognize methylated lysines and arginines through a conserved aromatic cage motif. A range of cognate targets have been identified including H3Rme2, H3K4me3, H3K36me3 and H4K20me2/3 [94]. Tudor domains consist of a single five-stranded β-barrel, and the TTD consists of two five-stranded β-barrels connected by a short (3–5 aa) linker. The knotted-Tudor contains two additional β-strands, one N-terminal and one C-terminal to the canonical five-stranded β-barrel. In addition to histone binding, three of these, the hPHF1 Tudor, 53BP1 TTD and scESA1 knotted-Tudor have been identified to interact with nucleic acids. These domains preferentially associate with dsDNA with moderate affinity (21 μM < K_d_ < 460 μM) [20,21,22,50], and the scESA1 knotted-Tudor was also found to bind with similar affinity to dsRNA. Notably, the Tudor family is the only example where DNA binding is consistently weaker than histone binding, at least for those studied to date. Though the Esa1 knotted-Tudor lacks an aromatic cage and does not associate with histones, the PHF1 Tudor binds H3K36me3 with K_d_ = 36 μM [48], and the 53BP1 TTD associates with H4K20me2 with K_d_ = 19.7 μM [49]. These domains are also unique in that binding is driven by largely hydrophobic rather than electrostatic contacts, as seen in see Figure 6. Binding pockets were all determined through NMR spectroscopy studies. The PHF1 Tudor and 53BP1 TTD recognize dsDNA using a similar interface consisting of largely hydrophobic residues in the β1-β2 loop and β3-β4 loops, as well as the β4-strand [20,21,22]. The Esa1 knotted-Tudor binds similarly, but utilizes the β2-β3 (instead of β1-β2) and β3-β4 loops for dsRNA interaction [50]. Though there is substantial overlap between the DNA and histone binding pockets, as seen in see Figure 6, PHF1 can bind both H3K36me3 peptide and dsDNA concomitantly. This suggests that a similar mechanism of interaction is occurring as is seen for the MSL3 CD, but that remains to be shown. However, PHF1 Tudor associates with H3K_C_36me3 nucleosomes with substantially higher affinity (K_d_ = 1.3 μM) than to dsDNA, H3K36me3 peptides or unmodified nucleosomes, consistent with a multivalent interaction [20].

The exact mechanism by which nucleic acid binding by these domains contributes to host protein/complex function is not yet clear. However, for 53BP1 and Esa1, binding has been shown to be functionally important. In the case of 53BP1, a protein involved in DNA-damage response and important for double-strand break repair, the dsDNA binding activity of the TTD was found to be critical for stimulating T4-ligase mediated non-homologous end joining [22]. Esa1 is the catalytic subunit of the NuA4 yeast acetyltransferase, which is important for several biological functions including DNA double-strand break repair, cell-cycle regulation and transcriptional control. Mutation of R62 in the nucleic acid binding pocket of the knotted-Tudor led to severe growth defects in the presence of genotoxic agents in yeast suggesting the importance of this activity in NuA4 function [50].

### 3.6. SANT Domains

SANT (Swi3, Ada2, NcoR, TFIIIB)/Myb domains consist of ~50 aa and are found in a number of nuclear proteins. These domains consist of a three α-helix bundle, stabilized by hydrophobic contacts, and generally connected by small 5–10 aa loops. A subset of SANT/Myb domains have been found to interact with unmodified histone H3 and H4 tails, reviewed in Reference [95]. However, their interaction with histones is still poorly understood. In addition, a large number SANT/Myb domains have been identified to bind dsDNA, which is much better studied. Several extensive reviews of DNA-binding SANT/Myb domains have been published [96,97,98,99,100]. Here, we focus on the SANT/Myb domains from c-Myb, coREST1, ISW1a, and *S. cerevisiae* Chd1, which are known to associate with histones and/or are part of histone modifying complexes and also represent each of the two known SANT/Myb domain modes of DNA binding. 

Two of the three c-Myb SANT/Myb domains (or repeats), one of the two coREST1 SANT domains, the ISW1a SANT domain, and the scChd1 SANT domain have all been shown to bind dsDNA [51,57,60,61]. The ISW1a and scChd1 SANT domains exist in the context of a HAND-SANT-SLIDE (HSS) combinatorial domain, where the HAND and SLIDE domains are additional DNA binding domains. Of these, the c-Myb repeats were shown to also interact with unmodified H3 and H4 tails [52,53,54], whereas co-REST1 SANT was found to lack histone binding activity [60,61]. Histone binding has not been tested for ISW1a and scChd1. Nucleic acid binding affinities have only been measured for the cMyb and coREST1 domains, and range between 10–81 μM. Notably, for cMyb, combining repeats 2 and 3 leads to a high affinity interaction with K_d_ = 3.8 nM. The cMyb domains have also been shown to have strong sequence specificity, with a consensus DNA-binding site of 5′-YAACNG-3′ where Y is a pyrimidine and N is any nucleotide [51,101]. 

**Figure 7:**
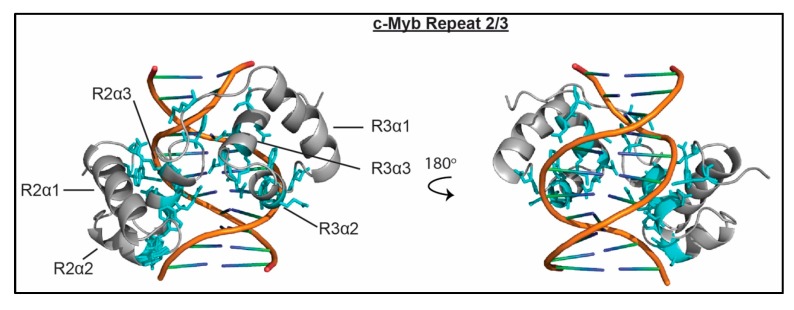
The molecular mechanism of SANT/Myb association with nucleic acid. The NMR structure of the murine cMyb SANT/Myb repeats 2 and 3 with a 26bp dsDNA containing the cognate sequence TAACGG (PDBID 1MSF) is shown. Residues important for complex formation are shown as cyan sticks.

These SANT/Myb domains utilize two common structural motifs to associate with DNA, either the α3 helix or the α1 helix. An NMR and crystal structure of cMyb repeats 2 and 3 with a 26 bp DNA sequence containing the core 5’-TAACGG-3’ consensus site have been solved [63,64]. The structure reveals that base-specific contacts are made by repeats 2 and 3, mediated by residues in the α3 helix of each domain. In addition to the base-specific contacts, several polar residues in both repeats 2 and 3 make contacts with the phosphate backbone of the DNA, as seen in Figure 7. Mutational analysis of coREST1 determined that, similar to c-Myb, basic residues in the α3 helix are important for DNA binding. However it is not yet clear if this leads to sequence specificity [60,61]. Crystal structures of the HSS domains of ISW1a and Chd1 reveal nearly identical mechanisms of SANT/DNA interaction, in which the α1 helix lies across the minor groove of the DNA and makes contacts with the phosphate backbone [55,58]. Recently, a 4.6 Å cryo-EM structure was solved of scChd1 in complex with a nucleosome containing 63 bp of linker DNA [102]. Notably, the scChd1 HSS binds the linker DNA in a manner that is identical to that of free DNA [58,102]. 

Nucleic acid binding by these domains is important in chromatin association and may play additional roles in the activity of the host protein/complex. The coREST1 protein is an accessory protein that regulates the activity of the LSD1 histone demethylase. Mutation of basic residues in the α3 helix resulted in decreased nucleosome binding by the LSD1-coREST1 complex and leads to a 5× decrease in coREST-mediated stimulation of LSD1 [60,61]. ISWI and Chd1 are both ATP-dependent remodeling proteins. Deletion of the dISWI SANT and SLIDE domain (which are highly homologous to scISW1a) was shown to significantly impair dISWI interaction with DNA and nucleosomes [56] and resulted in complete loss of ATPase and nucleosome remodeling activity of dISWI in vitro [56]. Similarly, mutation of basic residues in the scChd1 SANT domain significantly impaired DNA and unmodified nucleosome binding of scChd1, resulted in drastically reduced ATPase activity and nucleosome remodeling in vitro, and was unable to rescue a temperature sensitive yeast mutant lacking ISW1, ISW2, and Chd1 genes [59].

### 3.7. Integrated Domains

In addition to the individual domains known to associate with nucleic acids, there are two examples of integrated domains, which contain more than one reader domain, that have been found to associate with dsDNA. ZMYND8 and ZMYND11 are transcription co-repressors. Each contain an integrated structural and functional module of three linked reader domains, a PHD finger, BD, and PWWP domain, with an additional zinc finger (ZnF) connecting the BD and PWWP domain. In both proteins, the BD-ZnF-PWWP portion of the module forms a ‘V’ shape, where the ZnF sits between the BD and PWWP at the base of the V and forms extensive hydrogen bonds, salt bridges, and hydrophobic contacts with the two reader domains [69]. Although currently available ZMYND11 structures do not include the PHD finger, a ZMYND8 structure shows that the PHD finger, along with a helical linker, sits on the back side of the BD [62]. For each motif, both histone- and nucleic acid-binding activities have been observed. The ZMYND11 PHD-BD-ZnF-PWWP module specifically recognizes H3.3K36me3 [103], and ZMYND8 is specific for H3.3 containing K4me0/1 and K14ac, though binding has also been observed for acetylated H4 substrates [62,104,105]. Both have been found to bind to dsDNA [62,69,103]. Affinities have only been measured for ZYMND11, and are in the low micromolar range (K_d_ = 4.6–31 μM depending on sequence and conditions), which is comparable to the histone binding affinity (K_d_ = 56 μM). No preference for AT- or GC-rich DNA was observed among the sequences tested [69], but there appears to be preference for unmethylated over methylated DNA [103]. In an EMSA-based assay, the ZMYND11 BD-ZnF-PWWP binds to unmodified- and H3K_C_36me3-nucleosomes with apparent affinities of 0.95 μM and 0.16 μM, respectively, revealing a high affinity interaction and suggesting multivalent contacts with the histone tail and DNA [69].

**Figure 8:**
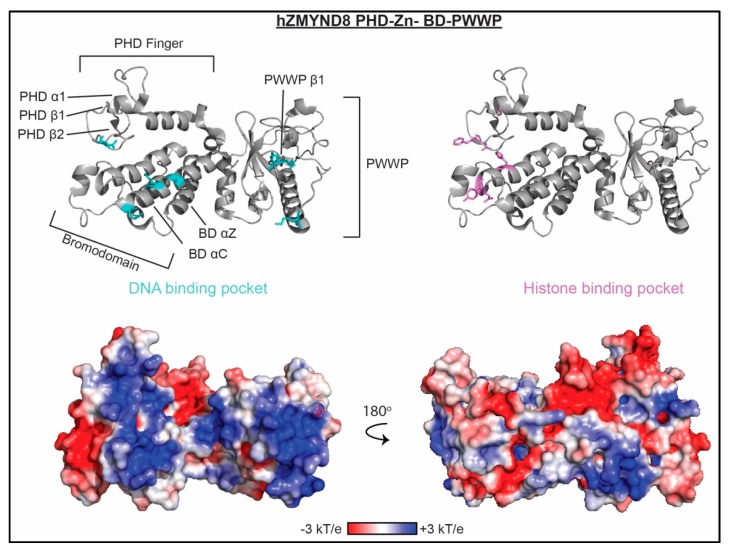
The molecular mechanism of an integrated domain association with nucleic acid. Residues determined to be important for binding nucleic acid as mapped by mutagenesis are shown as cyan sticks on ribbon representations of the ZYMD8 structure (PDBID 4COS). Residues important for histone binding are shown as violet sticks. Corresponding representation of the electrostatic surface (determined using the APBS plug-in in pymol) is shown in two orientations.

The DNA-binding residues were identified through mutational analysis and are adjacent to the histone binding pocket [62,69]. They are largely conserved between ZMYND11 and ZMYND8 and span all three domains, as seen in Figure 8. This includes regions on the PWWP that are comparable to the DNA-binding surfaces observed for the individual PWWP domains discussed above. In contrast, residues within the PHD finger and BD that are predicted to participate in interactions with DNA and are conserved between ZMYND11 and ZMYND8 are distinct from those observed for the individual domains discussed above. 

DNA binding is important for chromatin association of ZMYND8 and ZMYND11. Mutations to residues important for DNA-binding negatively affects the co-localization of ZMYND11 with H3K36me3-enriched regions in HeLa cells [69] and abrogates global chromatin association of ZMYND8 as assessed through chromatin fractionation assays [62]. In addition, recruitment of ZMYND8 to sites of DNA damage in U2OS cells is dependent on both histone- and DNA-binding abilities [62]. 

## 4. Auxiliary Nucleic Acid Binding Domains

For several of the reader domains harboring nucleic acid binding activity there are adjacent auxiliary nucleic acid binding motifs that coordinate with the reader domain to enhance DNA or RNA association through multivalent contacts. These are reminiscent of the N-terminal arms of homeodomains, which are important for DNA-binding affinity and specificity [106]. These often contain arginine residues, which are critical in association with DNA through contacts with the enhanced negative electrostatic potential of narrowed minor grooves of AT-rich sequences [107]. Although some of these motifs are detected by domain prediction algorithms, such as canonical AT-hooks, other motifs such as non-canonical AT-hooks or the N-terminal extension of SUV39H discussed below are not. Thus, a thorough analysis of the sequence surrounding the reader domain is important in order to identify these regions.

The CBX family of CDs all contain adjacent known or putative nucleic acid binding motifs that were originally identified through bioinformatics analysis [108]. These include AT-hooks, AT-like hooks, and extended AT-hooks. The canonical AT-hook is a small 10–15 amino acid motif containing a central GRP flanked by arginine and lysine residues [109]. This motif binds in the minor groove of DNA, with a moderate preference for the narrowed groove of AT-rich sequences. An extended AT-hook (eAT-hook) has also been described, with basic residues at a further distance from the core GRP than the canonical AT-hook, and has been found to preferentially bind RNA [110]. Finally, AT-like hooks (ATLs) contain a different core motif consisting of KRGPKP, whose preference for RNA or DNA in isolation is currently unknown. CBX2 contains an eAT-hook motif just C-terminal to the CD. CBX4/6/7/8 all have an ATL motif in the same position as the CBX2 eAT-hook, and CBX8 contains an additional ATL towards the C-terminus. The CBX2 eAT-hook and the CBX7 ATL have been shown to function with the adjacent CDs to associate with nucleic acids in a multivalent manner. The CBX2 CD and eAT-hook each preferentially associate with a 130nt ssRNA with K_d_ = 3.51 μM and K_d_ = 0.34 μM in 100 mM KCl, respectively [39], and the CD-eAT-hook together associate with a K_d_ = 0.17 μM, consistent with a multivalent binding mechanism [39]. In the case of CBX7, the CD alone preferentially binds ssRNA (discussed above). Intriguingly, though neither the CD nor the ATL bind with high affinity to dsDNA in isolation, the CD-ATL preferentially associates with dsDNA in 100 mM KCl [41]. Similar to the CBX proteins, the BRG1/BRM BD has an N-terminally adjacent AT-hook that binds DNA. This AT-hook does not alter the DNA binding mode of the BD, but the dual AT-hook-BD binds DNA tighter than the AT-hook or BD alone (K_d_ = 1.6 μM), consistent with a multivalent mode of binding, and imparts a moderate (~2-fold) specificity for AT-rich DNA [23].

PSIP1 carries an R/K-rich nuclear localization region and two AT-hooks C-terminal to the PWWP domain. These are further from the PWWP as compared to the other AT-hooks already discussed (>50 amino acids from the PWWP domain). In vitro binding assays and mutagenesis confirmed that the R/K-rich and AT-hook regions are important for DNA association, binding stronger than the PWWP domain to dsDNA [111]. In vivo, the R/K-rich region, AT-hooks and PWWP domain were all important for chromatin association. The PWWP contribution was suggested to be mainly attributable to histone binding, though notably, the full-length protein bound better to DNA in vitro than the construct containing the R/K-rich and AT-hook regions alone. Thus, the potential multi-valent activity of these regions is still not entirely clear.

The SUV39H1 protein contains a 41aa N-terminal extension (NTE) adjacent to the CD. This region is predicted to be folded, but has little structural similarity to any known protein motifs. The NTE was found to associate with a 19 nt ssRNA with an apparent K_d_ = 51 μM in isolation at 100 mM KCl [44]. When combined with the adjacent CD, the dual motif binds the same 19 nt ssRNA with an apparent K_d_ = 0.15 μM, consistent with a multivalent binding mechanism. Similarly, the addition of the C-terminal tail of PHF6 (predicted to be unfolded) to the ePHD2 leads to a roughly 2× increase in binding [27]. This suggests that the tail may function together with ePHD2 to bind DNA, and notably, this region is missing in several Börjeson-Forssman-Lehmann syndrome patients, indicating its functional importance.

## 5. Summary

In summary, it is now apparent that a number of histone reader domains harbor nucleic acid binding activity. We predict that additional members of these families, along with members of other reader domain families, will be found to have functionally important nucleic acid-binding properties in the future. Predicting these from sequence alone may prove to be difficult. However, some patterns are emerging regarding the protein interface utilized in nucleic acid binding. Especially if structural information is available, an analysis of the surface electrostatic potential could be predictive. However, as is clear from the Tudor domains, binding may also be driven by largely hydrophobic contacts, which is harder to predict. Continued studies into the mechanism by which these domains associate with nucleic acids is needed, particularly with regards to sequence specificity and structures of the complexes. In addition, as the functional importance of nucleic acid binding can be very difficult to assess based on in vitro structural and binding analysis alone, continued functional studies are critical. 

The ability to bind nucleic acids broadens the function of these domains, and highlights the need to characterize them within the broader context of the nucleosome and chromatin, with an eye towards this newly recognized function. The nucleic acid binding activity provides additional mechanisms for navigation and regulation of a complex chromatin landscape, which ultimately ensures the proper spatial and temporal control of chromatin structure and all DNA-templated processes. In addition, it provides novel therapeutic avenues for disruption of reader domain function. Given the strong therapeutic potential of many chromatin regulators, the continued characterization of nucleic acid binding function of these domains, especially the molecular basis of this function, are critical.

## Figures and Tables

**Table 1:** Nucleic acid binding domains. The domain, protein, and histone substrate is indicated. Dissociation constants for nucleic acids are shown if known, or indicated as (+) for detected binding with no determined affinity. Lack of binding is indicated by (−). NT indicates not tested. Nucleic acid substrates include single stranded (ss) or double-stranded (ds) RNA or DNA or nucleosomes.

Reader Domain	Protein	Organism	Histone Substrate	ssRNA	dsRNA	ssDNA	dsDNA	Nucleosome
BD	BRM [23]	*H. sapiens*	H3K14ac	NT	NT	NT	600 μM (10 bp)	NT
	BRG1 [23,24,25]	*H. sapiens*	H3K14ac	NT	NT	NT	+	NT
	BRDT1 BD1 [17]	*H. sapiens*	H4K5acK8ac	NT	NT	NT	52 μM (25 bp)	2 μM
PHD finger	BRPF2 PHD2 [26]	*H. sapiens*	none	NT	NT	9 μM (14 nt)	4 μM (14 bp)	NT
	PHF6 ePHD2 [27]	*H. sapiens*	none	NT	NT	NT	13 μM (14 bp)	NT
PWWP	DNMT3b [28]	*H. sapiens*	H3K36me3	NT	NT	NT	0.23 μM (30 bp)	NT
	Pdp1 [29]	*S. pombe*	H4K20me3	NT	NT	NT	4.9–5.3 μM (33 bp)	+
	HDGF [30,31]	*H. sapiens*	none	NT	NT	−	+	NT
	MSH6 [32,33]	*H. sapiens*	H3K36me3	NT	NT	2.5 μM (35nt)	0.13 μM (35 bp)	NT
	PSIP1/LEDGF [34,35]	*H. sapiens*	H3K36me3	NT	NT	NT	150 μM (10 bp), 1.6 μM (40 bp)	1.5 μM, 48 nM
CD/ Chromobarrel	MOF [36]	*D. melanogaster*	none	+	NT	NT	NT	NT
	MSL-3 [37,38]	*D. melanogaster*	H4K20me1	+	+	NT	0.4 μM (18 bp)	NT
	MSL-3 [37,38]	*H. sapiens*	H4K20me1	−	−	+	0.3 μM (18 bp)	NT
	CBX2 [18,39]	*H. sapiens*	H3K27me3	3.5 μM (130 nt)	NT	NT	8.1 μM (130 bp)	NT
	CBX4 [19]	*M. musculus*	H3K9me3	+	NT	NT	NT	NT
	CBX6 [19]	*M. musculus*	H3K27me3	+	NT	NT	NT	NT
	CBX7 [19]	*M. musculus*	H3K9me3	100 μM (500 nt)	+	NT	+	NT
	CBX7 [18,40,41]	*H. sapiens*	H3K9me3	51 μM (13 nt)	NT	NT	−	NT
	CBX8 [19]	*M. musculus*	H3K27me3	+	NT	NT	NT	NT
	SUV39H1 [42,43]	*M. musculus*	H3K9me3	0.35 μM (130 nt)	NT	NT	+	NT
	SUV39H1 [42,44]	*H. sapiens*	H3K9me3	2.3 μM (19 nt)	+	+	+	NT
	Clr4 [45,46]	*S. pombe*	H3K9me3	+	NT	+	+	NT
	Chp1 [45,46]	*S. pombe*	H3K9me3	3.9 μM (75 nt)	NT	+	+	NT
	MI-2 [47]	*D. melanogaster*	none	NT	NT	NT	+	+
Tudor	PHF1 [20,48]	*H. sapiens*	H3K36me3	NT	NT	−	201 μM (10 bp)	+
	53BP1 [21,49]	*M. musculus*	H4K20me2	NT	NT	NT	+	NT
	53BP1 [22,49]	*H. sapiens*	H4K20me2	NT	NT	+	+	NT
	Esa1 [50]	*S. cerevisiae*	none	NT	21–112 μM (varying)	NT	70–241 μM (varying)	NT
SANT/Myb	c-Myb R2R3 [51,52,53,54]	*H. sapiens*	unmod H3, unmod H4	NT	NT	NT	3.8 nM (16 bp)	+
	ISW1a [55]	*S. cerevisiae*	none	NT	NT	NT	+	+
	ISWI [56]	*D. melanogaster*	none	NT	NT	NT	+	+
	Chd1 [57,58,59]	*S. cerevisiae*	none	−	NT	−	+	+
	CoRest1 [60,61]	*H. sapiens*	none	NT	NT	NT	81 μM (18 bp)	+
Integrated Domains	ZMYND11	*H. sapiens*	H3K36me3	NT	NT	NT	31 μM (33 bp), 4.6 μM (22 bp)	0.95 μM (unmod), 0.16 μM (H3K36me3)
	ZMYND8 [62]	*H. sapiens*	H3K4me0, H3K14ac, H3K36me0	NT	NT	NT	+	NT
